# The ultimate radiochemical nightmare: upon radio-iodination of Botulinum neurotoxin A, the introduced iodine atom itself seems to be fatal for the bioactivity of this macromolecule

**DOI:** 10.1186/s13550-015-0083-5

**Published:** 2015-02-19

**Authors:** Janneke IM van Uhm, Gerard WM Visser, Marcel J van der Schans, Albert A Geldof, Eric JH Meuleman, Jakko A Nieuwenhuijzen

**Affiliations:** Department of Urology, VU University Medical Center, PO Box 7057, 1007 MB Amsterdam, The Netherlands; Department of Radiology and Nuclear Medicine, VU University Medical Center, Amsterdam, The Netherlands; TNO Defence, Security and Safety, Rijswijk, The Netherlands

**Keywords:** Botulinum neurotoxin A, Iodine-125, Radio-iodination, Monoclonal antibody, PET research

## Abstract

**Background:**

Botulinum neurotoxin A (BoNT-A) is a highly neurotoxic drug and frequently used in patients. Knowledge on the optimal way of administration of BoNT-A and its subsequent distribution is still rather limited. An accurate method for monitoring these processes might be the use of radiolabelled BoNT-A. In this paper, we report our feasibility study on labelling BoNT-A with high-dose iodine-125 (^125^I) via IODOGEN-coated BoNT-A method.

**Methods:**

Using cetuximab as model substrate for BoNT-A, a miniaturization of the IODOGEN-coated mAb method was developed with special attention to the minimum required amount of the oxidant IODOGEN, while the amount of substrate, reaction volume and reaction time were downsized. Labelling efficiency and radiochemical purity were determined by TLC, integrity by SDS-PAGE and HPLC and immunoreactivity by cell-binding assay. BoNT-A (50 μg) was labelled with ^125^I by coating with 2.5 μg IODOGEN, in a total reaction volume of 250 μL and a reaction time of 90 s. ^125^I-BoNT-A was purified by size exclusion chromatography (PD10 column) using ascorbic acid solution (5 mg/ml, pH = 5) as eluent. Quality analysis of ^125^I-BoNT-A was performed by an *in vitro* bladder strip model, an electrochemiluminescence assay and an Endopep assay.

**Results:**

Cetuximab (50 μg) labelling with ^125^I (15 to 150 MBq) resulted in a labelling efficiency of 70% to 80%, a radiochemical purity of >99%, an immunoreactivity of >95% and a retained integrity on SDS; HPLC analysis revealed partly affected integrity when 110 to 150 MBq ^125^I was used, i.e. when the averaged I/mAb molar ratio exceeded 3. Addition of HEPES (20 mM) and lactose (1.25%) (lyophilized BoNT-A contains HEPES and lactose) decreased the labelling efficiency to 44% to 54%. BoNT-A (50 μg) labelling with ^125^I (97.2 to 98.3 MBq) resulted in labelling efficiency of 51% to 52% with a radiochemical purity >98.5%, a specific activity of 150.5 to 152.9 MBq/nmol and an I/BoNT-A molar ratio of 1.86 to 1.90. The *in vitro* bladder strip model showed no bioactivity of ^125^I-BoNT-A when compared to unlabelled BoNT-A. The electrochemiluminescence and Endopep assay demonstrated around 10% and 15% bioactivity of ^125^I-BoNT-A compared to unlabelled BoNT-A, respectively. The remaining bioactivity correlates within the Poisson distribution with the amount of BoNT-A molecules that does not bear an iodine atom.

**Conclusions:**

BoNT-A was successfully radio-iodinated with an activity high enough to enable *in vivo* measurement of nanograms of BoNT-A, which could be used in studying optimization of administration techniques of BoNT-A. The bioactivity of a BoNT-A molecule is, however, lost upon the introduction of an iodine atom into the tyrosine moiety of this sensitive molecule.

## Background

Botulinum neurotoxin A (BoNT-A) was introduced for the treatment of overactive bladder (OAB) by Schurch et al. at the beginning of this century [1]. Since then, multiple clinical trials have proven BoNT-A to be an effective therapy for refractory OAB [2]. These clinical trials also demonstrated differences in efficacy and duration of the response. The most effective dose, volume of dilution, number and sites of injection required for effective treatment are not well known [3]. Notwithstanding evolving knowledge about the mechanism of action of BoNT-A on neurotransmitter release and receptor activation, the distribution of BoNT-A after intravesical injection remains largely unknown [4,5]. If the distribution in the bladder wall after administration of BoNT-A could be monitored, the differences in clinical effect among patients might be better understood. Besides, monitoring would enable investigation of the most optimal technique of administrating BoNT-A.

**Figure 1:**
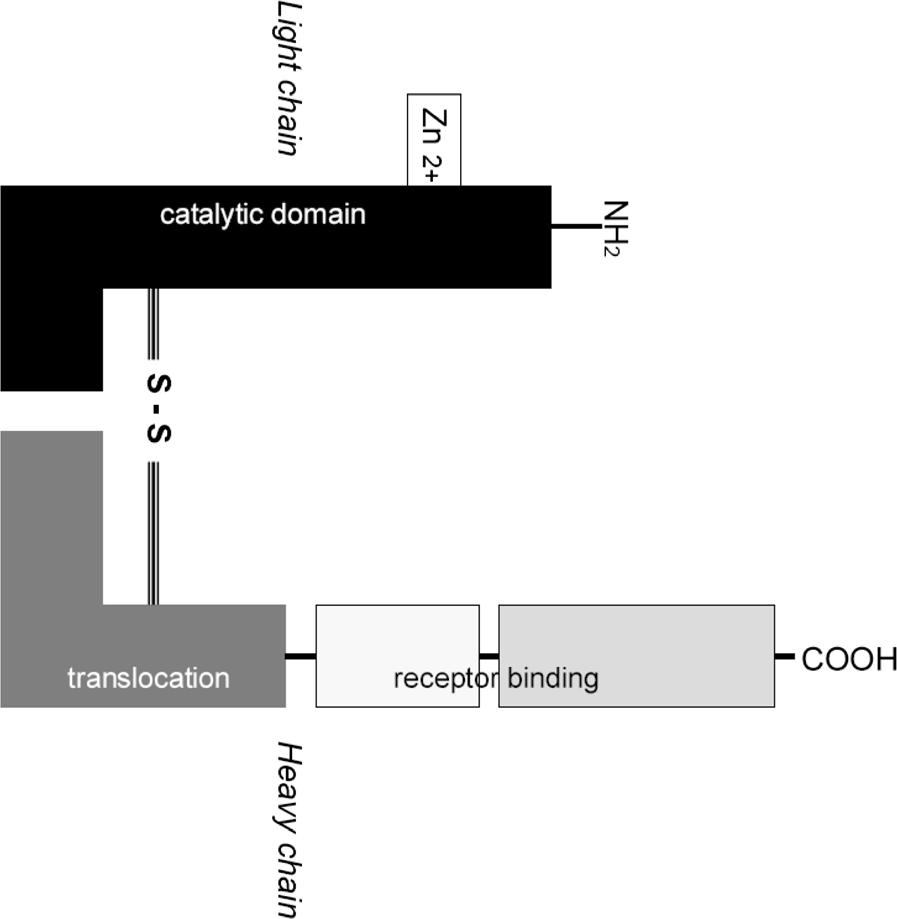
**BoNT-A molecule with heavy chain, light chain and disulphide connection.** The heavy chain (100 kD) is important for docking and endocytosis of BoNT-A in the nerve end terminal. In the nerve end terminal, the light chain (50 kD) cleaves SNAP-25 protein, which is essential in the fusion of acetylcholine vesicles with the nerve end membrane [17].

An accurate monitoring approach would be to label the 150 kD BoNT-A molecule (Figure 1) with a long living PET radioisotope like ^124^I or ^89^Zr, albeit with great demands on the conditions for the radiochemical reaction. Only nanograms of BoNT-A can be used in *in vivo* studies, due to its extremely high neurotoxicity (LD_50_ in humans is 1 ng/kg [6]). This implies that in order to enable detection with a PET camera, nanogram amounts of BoNT-A must be labelled with a high amount of radioactivity (high specific activity). Given a patient bladder injection dose of 2 ng, we calculated that 1 ng should contain at least 1 kBq, which corresponds with a specific activity of at least 150 MBq/nmol. To achieve this goal, a non-oxidative labelling route making use of an ester is rather problematic, since with tiny amounts of substrate hydrolysis of the ester is the main reaction at pH 9 to 9.5. Besides this, BoNT-A is nowadays supplied as lyophilized powder containing HEPES, so to reach pH 9 to 9.5, one has to break through the buffering capacity of HEPES in a controlled way as additional complicating factor. Therefore, the most straightforward route to arrive at 150 MBq/nmol seemed to be electrophilic radio-iodination, taking ^125^I as the research isotope for the ^124^I-PET isotope. Radio-iodination of BoNT-A has been reported earlier, but with the Bolton Hunter method, in accordance with what was stated above, only a specific activity of 8.1 MBq/nmol was obtained [7], while miscellaneous remaining bioactivity was obtained with chloramine-T [8].

The so-called IODOGEN-coated mAb method [9], originally developed for high-dose radio-iodination in a large reaction volume, has been shown to give results superior to the chloramine-T method and IODOGEN-coated vial method [9,10]. In this paper we report the development of a new radio-iodination method for obtaining optimal quality substrates in the 50-μg range, using cetuximab as model substrate. Next, our results with this miniaturized form of the IODOGEN-coated mAb method on the high-dose labelling of BoNT-A and its quality control are described.

## Methods

### Materials

The mAb cetuximab (Erbitux; 5 mg/mL) was purchased from Merck (Darmstadt, Germany) and was buffer exchanged on a PD10 column (GE Healthcare Life Sciences, Eindhoven, The Netherlands) to a solution of 0.9% NaCl before radiolabelling. ^125^I (12.95 GBq/mL in 0.1 M NaOH, pH 12 to 14, no-carrier added, specific activity 643.8 GBq/mg) was purchased from Perkin Elmer (Groningen, The Netherlands). 1,3,4,6-tetrachloro-3α, 6α-diphenyl-glycouril (IODOGEN, MW 432.1) was supplied by Pierce (Oud Beijerland, The Netherlands). Pure BoNT-A (vials of 100 μg, 150 kD) from *Clostridium botulinum*, lyophilized powder containing 20 mM HEPES and 1.25% lactose when reconstituted with 100 μL buffer, was supplied by Sigma-Aldrich (Zwijndrecht, The Netherlands). The reference BoNT-A was obtained from Metabiologics (Madison, WI, USA). The human squamous cell carcinoma cell line A431 was obtained from the American Type Culture Collection (ATCC number CRL-15555).

### Development of the labelling method

To avoid working routinely with 50 μg portions of extremely neurotoxic and expensive BoNT-A (LD_50_ in humans is about 75 ng [6]), developing and establishing the optimal method of labelling BoNT-A with ^125^I was done with cetuximab.

The coating approach was evaluated by several sets of labelling experiments within the following general frame: 1) every reaction was performed in a sterilized glass vial of 20 mL, which was wobbling continuously during the reaction; 2) the total reaction volume was 250 μL and consecutively consisted of X μL ^125^I in 0.1 M NaOH, 50 μL 0.5 M phosphate buffer (pH 7.4), 25 μL mAb solution (50 μg mAb, 2 mg/mL), 140-X μL 0.1 M phosphate buffer (pH 6.8) and 35 μL of IODOGEN/acetonitrile solution (1 mg/mL stock IODOGEN/acetonitrile solution was diluted to the desired amount of IODOGEN); 3) the reaction was started by IODOGEN/acetonitrile solution and abrogated with 100 μL ascorbic acid (25 mg/mL, pH 5); 4) labelling yields of all experiments were assessed with instant thin-layer chromatography (ITLC).

In the first set, labelling efficiency was determined upon decreasing the amount of IODOGEN (35, 20, 10, 5 and 2.5 μg), using 15 MBq ^125^I (75 MBq ^125^I/100 μL) and a reaction time of 180 s. In the second set, labelling kinetics was evaluated for 2.5 μg IODOGEN and a reaction time of 60, 90 and 180 s. In the third set, the amount of radioactivity of ^125^I was increased from 15 to 150 MBq. After PD10 column purification, the integrity was evaluated by high-performance liquid chromatography (HPLC) and sodium dodecylsulfate-polyacrylamide gel electrophoresis (SDS-PAGE) and the immunoreactivity by a cell-binding assay. In the fourth set, 50 μL HEPES (20 mM) and 12.5 μL lactose solution (50 mg/mL) was included in the reaction mixture of the cetuximab labelling. The influence of HEPES and lactose was studied, because lyophilized BoNT-A also contained these amounts of HEPES and lactose. The total dose of IODOGEN was either 2.5 μg in one portion, or it was added in two portions; 1.25 μg/17.5 μL IODOGEN/acetonitrile at the start of the reaction and a second portion after 45 s. Ten microlitres of bovine serum albumin (BSA pH 6.8; 50 mg/mL) was added to the reaction mixture before the PD10 column purification. Again, integrity was analysed by HPLC and SDS-PAGE. In the fifth set, 50 μg BoNT-A (50 μL) was labelled with 97.2 to 98.3 MBq ^125^I (10 μL) using the protocol described in Table 1. Special procedures were applied due to high neurotoxicity of BoNT-A, including the use of a biological safety cabinet, double rubber gloves, double-containment and contamination-free pipettes (Microman, Gilson, France). CAUTION: during try-outs with cetuximab, it was found that the advised safety measures (hypochlorite containing waste bin, spraying of vessels that came out of the biological safety cabinet with hypochlorite solution) completely destroyed the immunoreactivity of this mAb; So, these safety measures had to be left out. Geometry and calibration experiments were performed with ^125^I-labelled cetuximab to correct for self-absorption of the ^125^I-gamma’s in the various double containment vessels. Recovery of the ^125^I-BoNT-A mass was calculated as *50 μg* times *[MBq*^*125*^*I in product] / [MBq*^*125*^*I in starting vial times the labelling efficiency factor]*. ^125^I-BoNT-A and unlabelled BoNT-A were diluted in BSA/phosphate buffer (1 mg/mL) to a concentration of 1 μg/100 μL, and these batches were stored at −20°C.

**Table 1:** **Technical protocol** ^**125**^ **I labelling of BoNT-A according to IODOGEN-coated method**

**Step**	
1.	X μL ^125^I is added to wobbling reaction vial
2.	250 μL phosphate buffer (0.5 M, pH 7.4) is added
3.	50 μL BoNT-A/ phosphate buffer (=50 μg BoNT-A) is added^a^
4.	115-X μL phosphate buffer (0.1 M, pH 6.8) is added to adjust the reaction volume to 215 μL
5.	17.5 μL freshly prepared IODOGEN/acetonitrile solution (2.5 μg/35 μL) is added into the reaction mixture (start of reaction, *t* = 0)
6.	At *t* = 45 s, another 17.5 μL IODOGEN/acetonitrile solution (2.5 μg/35 μL) is added
7.	At *t* = 90 s, 100 μL ascorbic acid solution (25 mg/mL, pH 5.0) is added to stop the reaction and to reduce any formed S-Cl bonds
8.	At *t* = 4.30 min, 10 μL BSA (50 mg/mL) is added^b^
9.	At *t* = 5.30 min, samples are taken for ITLC for determination of the labelling efficiency
10.	At *t* = 10 min, 360 μL reaction mixture is transferred to a syringe connected to a filter^c^
11.	The reaction vial is rinsed by 640 μL phosphate buffer (0.1M, pH 6.8) and also transferred to the syringe connected to the filter^c^
12.	The combined solution in the syringe is filtered and purified on a PD10 column with ascorbic acid solution (5 mg/mL, pH 5.0) as eluent, collected fractions were 0.5 mL
13.	Fractions with highest amount of ^125^I-BoNT-A and the highest radiochemical purity are pooled
14.	Samples of the pooled ^125^I-BoNT-A (fractions 6, 7, 8) are taken for ITLC
15.	Final product is diluted by BSA (1 mg/mL) till a concentration of 1 μg/100 μL and stored at 20°C

*t* = time.

^a^Vial of 100 μg lyophilized BoNT-A powder is reconstituted with 100 μL phosphate buffer (0.1 M, pH 6.8).

^b^For encapsulation of the product to protect against radiation damage and to prevent absorption of material on the surface of the vials/syringes. Instead of BSA (bovine serum albumin), HSA (human serum albumin) or any other macromolecule can be used.

^c^Filter of 0.2 μm (Acrodisc Gelman Sciences, Ann Arbor, MI, USA).

### Quality analysis of mAb labelling

Conjugates were analysed by ITLC for labelling efficiency and radiochemical purity, by HPLC and SDS-PAGE followed by phosphor imager analysis for integrity and by a cell-binding assay for immunoreactivity. ITLC analysis of radiolabelled cetuximab was performed on silica gel-impregnated glass fibre sheets (PI Medical Diagnostic Equipment BV, Tijnje, The Netherlands), with citrate buffer (20 mM, pH 5.0) as the mobile phase. HPLC of the conjugates was performed on a JASCO Benelux BV HPLC (De Meern, The Netherlands) with a diode array detector system and an inline radiodetector (Raytest Isotopenmessgeräte GmbH, Straubenhardt, Germany) using a Superdex 200 10/300 GL size exclusion column (GE Healthcare Life Sciences, Eindhoven, The Netherlands). The eluent consisted of 0.05 M sodium phosphate/0.15 M sodium chloride plus 0.05% sodium azide (pH 6.8), and the flow was set at a rate of 0.5 mL/min. HPLC measurements were performed at *A* = 280 nm to measure mAb absorption. Gel electrophoresis was performed on a Pharmacia Phastgel System using 7.5% SDS-PAGE gel electrophoresis gels (Amersham Biosciences, Roosendaal, The Netherlands) under non-reducing conditions and analyzed on a phosphor imager (Molecular Dynamics, Zoetermeer, The Netherlands) and quantified with ImageQuant software. *In vitro* binding characteristics of radiolabelled cetuximab were determined in an immunoreactivity assay essentially as described before [11], using A431 cells fixed with 2% paraformaldehyde. Binding data were graphically analysed in a modified Lineweaver-Burk (double-reciprocal) plot, and the immunoreactive fraction was determined by linear extrapolation to conditions representing infinite antigen excess.

### Quality analysis of BoNT-A labelling

The bioactivity of ^125^I-BoNT-A was measured by an *in vitro* bladder strip model, an electrochemiluminescence (ECL) assay to measure the antibody-antigen detection on the heavy chain and an Endopep assay to measure the cleavage activity of the light chain.

The animal experiments were performed according to the Dutch National Institutes of Health principles of laboratory animal care and Dutch national (‘Wet op de dierproeven,’ Stb 1985, 336). The bladders of female Wistar rats (*n* = 2, 250 to 300 g, Harlan®, Horst, The Netherlands) were used for the *in vitro* bladder strip experiments previously described [12]. In short: two bladder strips of one rat bladder were mounted in two parallel organ baths. One strip was incubated in ^125^I-BoNT-A (0.3 mM) and one in unlabelled BoNT-A (0.3 mM). The contraction force of bladder strips due to electric field stimulation (EFS) was measured before and after incubation. The second quality analysis of ^125^I-BoNT-A was by ECL assay [13]. The ECL assay is based on antibody docking of the heavy chain of BoNT-A, which is in this case the antigen. ECL 96-well plates were coated with BoNT-A capture antibodies. The unlabelled BoNT-A, reference BoNT-A (TNO, Rijswijk, The Netherlands) and ^125^I-BoNT-A were diluted in concentrations of 20, 5, 1.25, 0.31, 0.078, 0.02 and 0.005 ng/mL. These various concentrations were pipetted in the wells, and the plate was stirred and washed. The wells were filled with MSD-labelled detection antibody, stirred and washed. Finally, the wells were filled with READ buffer and the signals could be read with the ECL instrument (PR-100, Meso Scale Discovery, Gaithersburg, MD, USA).

The third quality analysis of ^125^I-BoNT-A was by Endopep assay [14]. This assay is based on the cleavage activity of the light chain. Unlabelled BoNT-A, reference BoNT-A and ^125^I-BoNT-A were diluted in concentrations of 0.5, 0.2, 0.1, and 0.02 ng/μL. These samples were mixed with 20 μL Endopep reaction buffer (20 mM HEPES pH 7.3, 200 μM ZnCl_2_, 1 mg/mL BSA and 10 mM DTT). Substrate peptide (KGSNRTRIDQGNQRATR-NLeu-LGGK); 10 μL, 100 μM) was added, and the mixture was incubated at 36°C overnight. The activity of the light chain of BoNT-A was measured in a capillary electrophoresis instrument with laser-induced fluorescence (LIF) detection (Beckman Instruments, Fullerton, CA, USA). The excitation wavelength was 488 nm, and the emission wavelength was 520 nm. The total length of the fused silica capillary (i.e. 75 μm, PolyMicro, Phoenix, AZ, USA) was 67 cm; effective length to the detector was 60 cm. The separation voltage was 15 kV. Samples were introduced by pressure injection for 5 s at 0.5 psi. The running buffer consisted of 60 mM sodium hydroxide and 18 mM phosphoric acid, pH 12.

## Results

### Radiolabelling of mAb and BoNT-A

**Figure 2:**
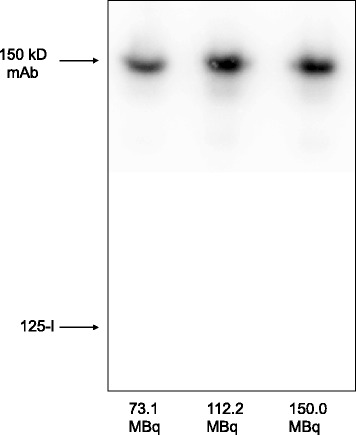
**Phosphor imager picture of SDS-PAGE gel of the product from the** ^**125**^ **I-cetuximab labelling reaction.** The ^125^I-cetuximab labelling reaction with 73.1, 112.2 and 150.0 MBq with miniaturized IODOGEN-coated mAb method demonstrating unaffected integrity with respect to the molecular weight. Conditions: 50 μg (0.33 nmol) cetuximab, 2.5 μg (5.7 nmol) IODOGEN, 250 μL reaction volume, 90-s reaction time.

**Figure 3:**
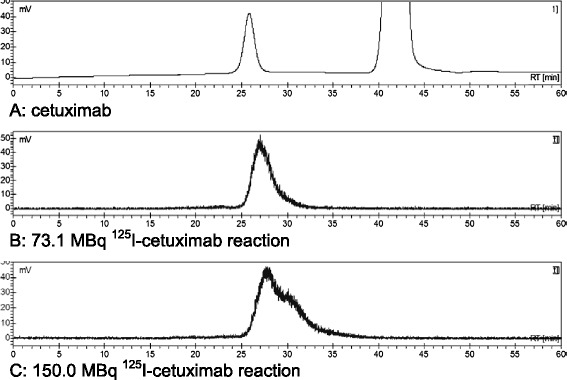
**HPLC chromatograms of** ^**125**^ **I-cetuximab after PD10 column purification.** Channel **A** shows the UV absorption of cetuximab at 280 nm at a retention time of 26 min (large peak at 42 min is from ascorbic acid). Channels **B** and **C** represent the radioactive signal of ^125^I-cetuximab from the 73.1 MBq and 150.0 MBq reaction demonstrating retained and impaired integrity, respectively.

Labelling of 50 μg cetuximab with 15 MBq ^125^I using IODOGEN-coated mAb method in 250 μL reaction volume and reaction time of 90 s resulted in a labelling yield of 70% to 80%. There was no difference in labelling yield by using 35 μg till 2.5 μg IODO-GEN. Also, no substantial increase in labelling yield was observed for a reaction time above 90 s. The same conditions and an increase of amount of radioactivity to 150 MBq resulted in a labelling yield of 71% to 81% (Table 2). ITLC showed that the radiochemical purity of the products was ≥99% after size exclusion chromatography (PD10). The phosphor imager analysis of the SDS-PAGE gel of the 73.1 MBq, 112.2 MBq and 150.0 MBq reactions is depicted in Figure 2. It revealed unaffected integrity with respect to molecular weight: only the presence of the major 150 kD BoNT-A band and a low amount (0.1% to 0.4%) of free iodine was observed. The HPLC analysis resulted in an unaffected integrity up to the 73.1 MBq reaction and a partly impaired integrity increasing for the 112.2 MBq and 150.0 MBq reactions (shown for 73.1 and 150.0 MBq reaction in Figure 3). Since the molecular weight was unaffected (Figure 2) and any formed S-Cl bonds are reduced by ascorbic acid, this remarkable finding indicates that a too heavy load of iodine atoms induces a conformational change of the mAb molecule. It was, therefore, decided that BoNT-A should not be labelled with an I/BoNT-A molar ratio higher than two, even though the immunoreactivity of ^125^I-cetuximab did not change by increasing the amount of radioactivity (Table 2). To determine the influence of HEPES and lactose on the labelling reaction, both were added to the reaction mixture. Lactose had no effect on the labelling yield, but addition of HEPES induced a decrease in labelling yield from 80.7% to 43.5% (Table 3). The addition of IODOGEN/acetonitrile reagent in two portions increased the labelling efficiency from 43.5% to 51.8%. The radiochemical purity was >98.5%. As before, the product with ^125^I/mAb molar ratio of 2.3 showed immunoreactivity of ≥95% and unaffected integrity of the product.

**Table 2:** **Results of iodination of cetuximab with increasing amount of** ^**125**^ **I**

**Cetuximab (μg)**	^**125**^ **I** **(MBq)**	**Labelling efficiency (%)**	**Radiochemical purity (%)**	**Immunoreactivity (%)**	^**125**^ **I/mAb molar ratio** ^a^
50	15.0	71.4	99.1	95.2	0.40
50	48.4	75.2	99.7	ND	1.36
50	73.1	81.9	99.3	96.9	2.29
50	112.2	72.9	99.8	ND	3.05
50	150.0	77.0	99.4	96.8	4.30

Conditions: 2.5 μg IODOGEN, 250 μL reaction volume and 90-s reaction time. ND = not determined.

^a^100 MBq ^125^I corresponds with 1.24 nmol I atoms.

**Table 3:** **Results of iodination of cetuximab with addition of HEPES and/or lactose and iodination of BoNT-A**

**Product (50 μg)**	**HEPES (μmol)**	**Lactose (mg)**	**IODOGEN (μg)**	^**125**^ **I (MBq)**	**Labelling efficiency (%)**	^**125**^ **I/product molar ratio**
Cetuximab	0	0	2.5	19.6	80.7	0.59
Cetuximab	0	0.625	2.5	19.3	78.3	0.56
Cetuximab	1	0	2.5	15.7	43.5	0.25
Cetuximab	1	0.625	2.5	30.2	43.5	0.49
Cetuximab	1	0.625	1.25 + 1.25	15.6	50.2	0.29
Cetuximab	1	0.625	1.25 + 1.25	93.4	51.8	1.80
Cetuximab	1	0.625	1.25 + 1.25	146.2	43.0	2.34
BoNT-A	-^a^	-^a^	1.25 + 1.25	97.2	51.5	1.86
BoNT-A	-^a^	-^a^	1.25 + 1.25	98.3	51.8	1.90

Conditions: 2.5 μg IODOGEN in 35 μL IODOGEN/acetonitrile added at *t* = 0 or 1.25 μg IODOGEN in 17.5 μL IODOGEN/acetonitrile added at *t* = 0 and at *t* = 45. Other conditions: 250 μL reaction volume and 90-s reaction time. Radiochemical purity was >98.5% in all experiments.

^a^Reconstituted BoNT-A contained HEPES (20 mM) and lactose (1.25%); this was not purposely added.

**Figure 4:**
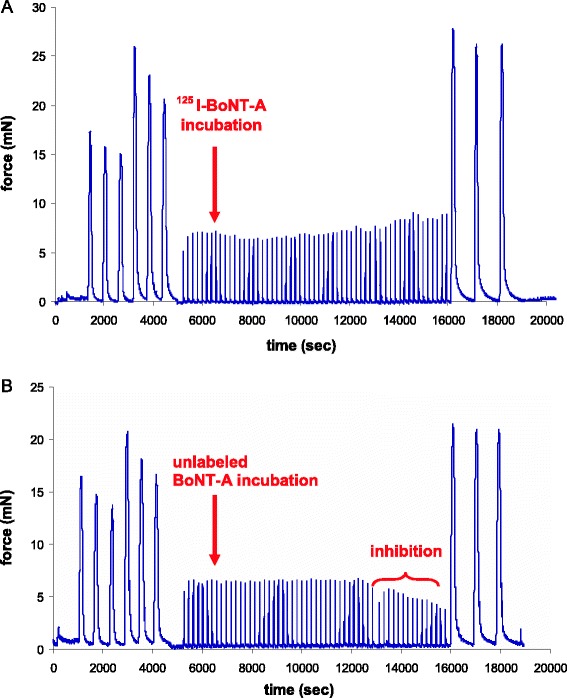
**The results of the rat bladder strip** ***in vitro*** **model.** Rat bladder strip mounted in two parallel organ baths and incubated in ^125^I-BoNT-A **(A)** showing no inhibition of contraction force induced by electrical field stimulation (EFS) in time and unlabelled BoNT-A **(B)** showing inhibition of contraction force induced by EFS in time. Both strips are simultaneously stimulated by potassium chloride (KCl; three times), carbachol (CCh; three times), EFS (repetitive) and CCh (three times). The CCh-induced strip contraction before and after incubation is a viability check of the strip during the experiment.

**Figure 5:**
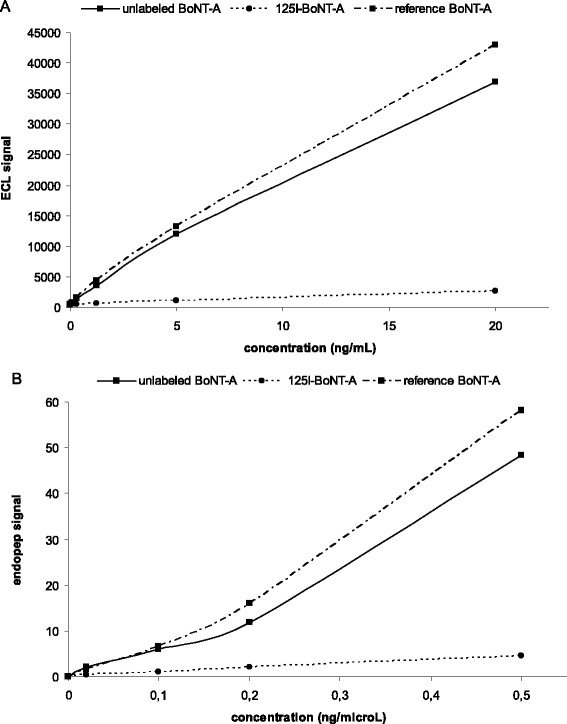
**The results of the ECL and Endopep assay.** The ECL signal of unlabelled BoNT-A, ^125^I-BoNT-A and reference BoNT-A **(A)**. The Endopep signal of unlabelled BoNT-A, ^125^I-BoNT-A and reference BoNT-A **(B)**.

BoNT-A (50 μg) labelling with ^125^I (97.2 to 98.3 MBq) by the developed method resulted in a labelling efficiency of 51.5% to 51.8% and a radiochemical purity of 98.9% (Table 3). The specific activity was 150.5 to 152.9 MBq/nmol BoNT-A. Unlike unlabelled BoNT-A (0.3 mM), ^125^I-BoNT-A (0.3 mM) did not result in inhibition of bladder strip contraction induced by electrical field stimulation (Figure 4). The ECL signal from ^125^I-BoNT-A was about 10% of the signal of unlabelled BoNT-A and of the reference-BoNT-A (Figure 5A). The Endopep signal from ^125^I-BoNT-A was about 15% of the Endopep signal of unlabelled BoNT-A and of the reference-BoNT-A (Figure 5B).

## Discussion

In this article, a novel method is described for high-dose ^125^I labelling of macromolecules on microgram scale. A special feature of this miniaturized form of the IODOGEN-coated mAb method is the low amount (5.7 nmol) of the oxidizing agent IODOGEN required to arrive at efficient labelling yields. For cetuximab labelling, yields of 70% to 80% were obtained; in the presence of HEPES and lactose, the yields were 43% to 52%. Conjugates with high specific activity and with full retention of immunoreactivity and integrity were produced, provided the averaged I/mAb molar ratio was kept below 3. The labelling of BoNT-A to a level of specific activity necessary for sensitive detection by PET, i.e. 1 kBq/ng BoNT-A, was accomplished by this IODOGEN-coated BoNT-A method in a yield of 51% to 53%. The bioactivity of ^125^I-BoNT-A, however, was found to be seriously impaired when compared to unlabelled BoNT-A in three different assays. In an *in vitro* rat bladder strip model, contraction inhibition was not observed in case of ^125^I-BoNT-A. A reason could be a lower total amount of ^125^I-BoNT-A or a change of bioactivity of ^125^I-BoNT-A. To rule out the possibility of a concentration mishap, a batch of dry powder BoNT-A (100 μg) was dissolved in 100 μL phosphate buffer, in a weighing controlled way. Again controlled by weighing, 50 μL of this solution was used for a labelling reaction whereas the remaining 50 μL was diluted to a concentration of 1 μg/100 μL unlabelled BoNT-A and compared with a reference sample of BoNT-A (TNO) by determining the heavy and light chain bioactivity by antibody-antigen detection with ECL - and Endopep assay. Both assays gave for the unlabelled BoNT-A similar results as the reference BoNT-A, indicating that no dissolution mishap had occurred and that the amount of BoNT-A at the start of the labelling reaction was indeed the anticipated 50 μg. At the same time, the results of the ECL assay and the Endopep assay therefore implied ^125^I-BoNT-A had lost a great deal of its bioactivity.

In the 50 μg BoNT-A situation, the IODOGEN/BoNT-A molar ratio was 17; and under these conditions, no detectable harm to cetuximab was observed with respect to immunoreactivity or integrity. This and the fact that in 1983 Williams et al. [8] found remaining bioactivity even at chloramine-T/BoNT-A molar ratios of 200 to 300 make that damage from chemical oxidation cannot be the main reason for the huge loss of bioactivity. Also, the most effective chemoprotection against radiation damage (no Cl^−^ containing PBS but phosphate buffer [15], addition of ascorbic acid and BSA [9]) before, during and after PD10 column purification of the ^125^I-BoNT-A was implemented, ruling out radiation damage, if any, as possible reason.

Hence, we arrive at the conclusion that the main loss of bioactivity does not originate from oxidative or radiation damage but from the introduction of the iodine atom itself, i.e. from the introduction of an apolar heavy atom combined with the effect of the decrease of the pKa of the iodinated phenol moiety from 10.0 to 8.9. A strong extra argument in favour of this conclusion can be derived from the fact that the averaged I/BoNT-A molar ratio of our ^125^I-BoNT-A with specific activity of 150.5 to 152.9 MBq/nmol is 1.86 to 1.90 (Table 3). This means that within the Poisson distribution, around 15% of the BoNT-A molecules do not bear an iodine atom, a percentage that fairly correlates with the remaining bioactivity in the ECL and Endopep assay. Also, the most optimal findings of Williams et al. [8], obtained with a chloramine-T/BoNT-A molar ratio of 23 to 35, fit with this concept. Their ^125^I-BoNT-A products with a specific activity of 700 to 712 and 1,532 to 1,750 Ci/mmol correlate with an averaged I/BoNT-A molar ratio of 0.32 and 0.75, respectively, or within the Poisson distribution with a zero iodination fraction of around 73% and 47%. This corresponds remarkably well with the reported remaining bioactivity of around 85% and 60%, respectively, bearing in mind that in those days the starting product contained a certain amount of contaminating protein which was bio-inactive at forehand.

Labelling with ^124^I requires the addition of 200 pmol iodine carrier to get efficient yields [16]. Our established specific activity results with ^125^I-BoNT-A imply that this carrier aspect will not lead to a labelling problem. ^124^I-BoNT-A might therefore contribute to setting up a protocol for optimal dose injection and quantification in the bladder wall in a selected panel of patients. It is very uncertain, however, whether ^124^I-BoNT-A is representative for the distribution of BoNT-A through the bladder wall after intravesical injection. The heavy chain is important for docking and endocytosis of BoNT-A in the nerve end terminal, while the light chain cleaves SNAP-25 protein in the nerve end terminal, SNAP-25 being essential in the fusion of acetylcholine vesicles with the nerve end terminal membrane [17]. When an iodine atom on essential tyrosine moieties blocks these subtle processes, it means that the PET signal of the ^124^I-BoNT-A molecule does not reflect the behaviour of an unlabelled BoNT-A molecule at cellular level.

On the basis of earlier studies with mAbs, we could state (1) that one cannot unlimitedly change the positive surrounding of lysine groups (for mice, the limit was eight groups; for patients, four groups before seriously affecting pharmacokinetics) [18], and (2) that the introduction of a large hydrophobic group like IRDyeCW800 is limited to 1 [19]. The findings in this study allow us to add a third statement, namely that the more specific the macromolecule the less alterations, even small ones, are permitted.

## Conclusions

We have developed a mild reliable and efficient electrophilic radio-iodination procedure for GMP-compliant labelling of macromolecules on microgram scale. The integrity of a mAb molecule becomes impaired when on average the number of introduced iodine atoms is ≥3. For the very sensitive macromolecule BoNT-A, however, a single introduced iodine atom already destroys the bioactivity.

## References

[1] Schurch B, Stohrer M, Kramer G, Schmid DM, Gaul G, Hauri D (2000). Botulinum-A toxin for treating detrusor hyperreflexia in spinal cord injured patients: a new alternative to anticholinergic drugs? Preliminary results. J Urol..

[2] Duthie JB, Vincent M, Herbison GP, Wilson DI, Wilson D (2011). Botulinum toxin injections for adults with overactive bladder syndrome. Cochrane Database Syst Rev.

[3] Mangera A, Apostolidis A, Andersson KE, Dasgupta P, Giannantoni A, Roehrborn C (2014). An updated systematic review and statistical comparison of standardised mean outcomes for the use of botulinum toxin in the management of lower urinary tract disorders. Eur Urol..

[4] Apostolidis A, Dasgupta P, Fowler CJ (2006). Proposed mechanism for the efficacy of injected botulinum toxin in the treatment of human detrusor overactivity. Eur Urol..

[5] Coelho A, Cruz F, Cruz CD, Avelino A (2012). Spread of onabotulinumtoxinA after bladder injection. Experimental study using the distribution of cleaved SNAP-25 as the marker of the toxin action. Eur Urol.

[6] Gill DM (1982). Bacterial toxins: a table of lethal amounts. Microbiol Rev..

[7] Ravichandran E, Gong Y, Al Saleem FH, Ancharski DM, Joshi SG, Simpson LL (2006). An initial assessment of the systemic pharmacokinetics of botulinum toxin. J Pharmacol Exp Ther..

[8] Williams RS, Tse CK, Dolly JO, Hambleton P, Melling J (1983). Radioiodination of botulinum neurotoxin type A with retention of biological activity and its binding to brain synaptosomes. Eur J Biochem..

[9] Visser GW, Klok RP, Gebbinck JW, Ter LT, van Dongen GA, Molthoff CF (2001). Optimal quality (131)I-monoclonal antibodies on high-dose labeling in a large reaction volume and temporarily coating the antibody with IODO-GEN. J Nucl Med..

[10] Tran L, Baars JW, Maessen HJ, Hoefnagel CA, Beijnen JH, Huitema AD (2009). A simple and safe method for 131I radiolabeling of rituximab for myeloablative high-dose radioimmunotherapy. Cancer Biother Radiopharm..

[11] Lindmo T, Boven E, Cuttitta F, Fedorko J, Bunn PA (1984). Determination of the immunoreactive fraction of radiolabeled monoclonal antibodies by linear extrapolation to binding at infinite antigen excess. J Immunol Methods..

[12] Van Uhm JIM, Beckers GM, van der Laarse WJ, Meuleman EJ, Geldof AA, Nieuwenhuijzen JA (2014). Development of an in vitro model to measure bioactivity of botulinum neurotoxin A in rat bladder muscle strips. BMC Urol..

[13] Rivera VR, Gamez FJ, Keener WK, White JA, Poli MA (2006). Rapid detection of Clostridium botulinum toxins A, B, E, and F in clinical samples, selected food matrices, and buffer using paramagnetic bead-based electrochemiluminescence detection. Anal Biochem..

[14] Kalb SR, Moura H, Boyer AE, McWilliams LG, Pirkle JL, Barr JR (2006). The use of Endopep-MS for the detection of botulinum toxins A, B, E, and F in serum and stool samples. Anal Biochem..

[15] Perk LR, Vosjan MWD, Visser GWM, Budde M, Jurek P, Kiefer GE (2010). p-Isothiocyanatobenzyl-desferrioxamine: a new bifunctional chelate for facile radiolabeling of monoclonal antibodies with zirconium-89 for immuno-PET imaging. EJNMMI.

[16] Tijink BM, Perk LR, Budde M, Stigter-van Walsum M, Visser GWM, Kloet RW (2009). 124I-L19-SIP for immuno-PET imaging of tumor vasculature and guidance of 131I-L19-SIP radioimmunotherapy. EJNMMI..

[17] Dolly JO, Aoki KR (2006). The structure and mode of action of different botulinum toxins. Eur J Neurol..

[18] Van Gog FB, Visser GW, Stroomer JW, Roos JC, Snow GB, van Dongen GA (1997). High dose rhenium-186-labeling of monoclonal antibodies for clinical application: pitfalls and solutions. Cancer.

[19] Cohen R, Stammes MA, De Roos IHC, Stigter-Van Walsum M, Visser GWM, Van Dongen GAMS (2011). Inert coupling of IRDye800CW to monoclonal antibodies for clinical optical imaging of tumor targets. EJNMMI Res..

