# Erratum to: The ultimate radiochemical nightmare: upon radio-iodination of Botulinum neurotoxin A, the introduced iodine atom itself seems to be fatal for the bioactivity of this macromolecule

**DOI:** 10.1186/s13550-015-0108-0

**Published:** 2016-01-26

**Authors:** Janneke van Uhm

**Affiliations:** Vu Medical Center, Department of Urology, Amsterdam, Netherlands

## Erratum

After publication of the manuscript [1], it emerged there have been a mistake in Table 1. At step 2, 50 μL phosphate buffer (0.5 M, pH 7.4) is added in stead of 250 μL which is described.

We apologise for any inconvenience.
